# Synaptic and Circuit Mechanisms Shaping Neurodevelopmental and Psychiatric Outcomes Associated with 16p11.2 Copy Number Variation

**DOI:** 10.3390/genes17060716

**Published:** 2026-06-21

**Authors:** Alžbeta Námešná, Jasmine Pickford, Jeremy Hall, Marianne van den Bree, Luke Tait, Lawrence S. Wilkinson, Matt W. Jones

**Affiliations:** 1 Neuroscience and Mental Health Innovation Institute, School of Medicine, Cardiff University, Cardiff CF24 4HQ, UK or namesnaa@cardiff.ac.uk (A.N.);; 2 Division of Psychological Medicine and Clinical Neurosciences, School of Medicine, Cardiff University, Cardiff CF24 4HQ, UK; 3 School of Psychology and Neuroscience, University of Bristol, Bristol BS8 1TD, UK; 4 Department of Psychiatry, Medical Sciences Division, University of Oxford, Oxford OX3 7JX, UK; 5 MRC Centre for Neuropsychiatric Genetics and Genomics, Cardiff University, Cardiff CF24 4AG, UK; 6 Cardiff University Brain Research Imaging Centre, School of Psychology, Cardiff University, Cardiff CF24 4HQ, UK; 7 School of Mathematics, Cardiff University, Cardiff CF24 4AG, UK; 8 School of Psychology, Cardiff University, Cardiff CF24 4AG, UK

**Keywords:** copy number variants, 16p11.2, deletions, gene dosage, synaptic plasticity, neurodevelopmental disorders, neuropsychiatric disorders, brain development

## Abstract

Copy number variants (CNVs) are genomic rearrangements that carry a substantial risk for neurodevelopmental and neuropsychiatric disorders. Among these, recurrent deletions and duplications at the 16p11.2 locus are robustly associated with autism spectrum disorders, schizophrenia, epilepsy, and related conditions, yet also display marked variability in penetrance and phenotypic expression. Accumulating evidence indicates that 16p11.2 gene dosage influences multiple stages of brain development, from early progenitor dynamics and neuronal migration to synaptic formation, refinement, and plasticity. However, how disruptions across these processes are integrated over time, and how they relate to the observed variability and incomplete penetrance, remains poorly understood. In this review, we summarize the current evidence on the impact of 16p11.2 CNVs on brain development, focusing on cellular and circuit-level processes that shape neural connectivity. We discuss how gene dosage imbalance influences early developmental trajectories, synaptic formation and pruning, interneuron maturation, and activity-dependent plasticity, and consider how these processes interact across developmental stages. We suggest a conceptual framework wherein 16p11.2 CNVs do not impose fixed pathogenic outcomes, but rather they contribute towards developmental constraints that shape the timing and stability of neural circuit development. Consequently, these constraints increase vulnerability to neurodevelopmental and psychiatric outcomes in a context-dependent manner.

## 1. Introduction

Neurodevelopmental and neuropsychiatric disorders (NDDs and NPDs, respectively) are a major global health burden and a leading cause of long-term disability [1]. Conditions such as autism spectrum disorder (ASD), intellectual disability (ID), schizophrenia (SCZ), and attention-deficit/hyperactivity disorder (ADHD) are highly prevalent in the general population (ranging from 0.28 to 11% depending on the condition [2,3]) and clinically heterogeneous, both within diagnostic categories and across conditions [4]. Advances in our understanding of the neurobiology underlying these conditions are challenging traditional diagnostic boundaries [5]. Increasing evidence suggests that, despite differences in behavioral phenotypes and ages of onset, these disorders may share common developmental origins in disrupted brain maturation [6]. In particular, disturbances to the formation and refinement of neural circuits during early development are thought to confer vulnerability to a broad spectrum of cognitive, behavioral and psychiatric outcomes [7,8].

Copy number variants (CNVs) are segmental micro-deletions or -duplications of genomic material and represent a major source of structural genetic variation in the human genome [9]. CNVs vary widely in size, from a few kilobases to several megabases. Large CNVs are surprisingly common in the general population: approximately 5–10% of individuals carry a CNV ≥500 Kb, and approximately 1–2% carry a CNV ≥1 Mb in size [9,10]. However, most such variants are extremely rare at the locus level, and only a small subset, termed “recurrent” CNVs, which occur at specific genomic hotspots, are consistently associated with high neurodevelopmental risk [11,12]. Indeed, over the past two decades, rare (<1% of the population) large (≥500 Kb) CNVs, such as 22q11.2 and 1q21.1 microdeletions or 7q11.23 microduplication, have emerged as important genetic risk factors for a broad range of neurodevelopmental and neuropsychiatric disorders, including autism spectrum disorder, intellectual disability, epilepsy, and schizophrenia [13,14,15,16,17,18,19,20,21,22].

CNVs may arise de novo (i.e., they are not present in the somatic cells of the parents but occur spontaneously during meiosis and formation of the parental gametes) or be inherited. A subset recur at specific loci due to susceptibility to genomic rearrangements, such as non-allelic homologous recombination (NAHR), giving rise to so-called recurrent CNVs [9,23]. Recurrent pathogenic CNVs are typically observed in the heterozygous state, with deletions or duplications affecting one chromosomal copy while the other remains intact. Large case–control studies have consistently demonstrated an increased burden of rare CNVs in clinically ascertained cohorts relative to the general population. These studies have also identified multiple recurrent loci that confer substantial risk across diagnostic categories, including physical health conditions [14,19,24,25,26,27,28,29,30]. Notably, pleiotropy is a defining feature of CNV-associated risk. A single CNV may predispose to multiple clinical outcomes, while similar neurodevelopmental phenotypes can arise from disruptions at distinct genomic loci. This lack of diagnostic specificity suggests that pathogenic CNVs may converge on shared developmental and cellular pathways, rather than encoding disorder-specific mechanisms [4,31]. This convergence has motivated a shift beyond locus-by-locus descriptions toward mechanistic frameworks linking altered gene dosage to disrupted brain development and neural circuit function.

The 16p11.2 locus has emerged as one of the most penetrant and clinically relevant CNVs associated with a range of NDDs and NPDs [32]. Located on the short (p) arm of chromosome 16, this region contains two recurrent CNV hotspots, termed the proximal and distal 16p11.2 loci. Both regions are flanked by clusters of highly homologous segmental duplications, termed breakpoints (BPs), which predispose them to recurrent rearrangements via NAHR during meiosis [33]. The proximal 16p11.2 CNV occurs between BP4-BP5, from genomic coordinates 29.5 Mb to 30.1 Mb (~593 Kb in size), whereas the distal CNV is smaller, located between BP2 and BP3 at coordinates from 28.7 Mb to 29 Mb (~220 Kb size). Although CNVs at both loci are associated with elevated neurodevelopmental risk [34], the proximal 16p11.2 locus is more strongly implicated in a broad range of brain-related phenotypes, and thus will be the focus of the present review.

The proximal 16p11.2 CNV encompasses around 29 protein-coding genes, in addition to several non-coding regulatory elements [35]. Both deletions and duplications of this interval occur (estimated population prevalence 0.03–0.05% [36,37]), and each confers increased risk for neurodevelopmental and neuropsychiatric outcomes. Populations carrying either deletion or duplication of the proximal 16p11.2 locus show elevated rates of ASD [13,14,38,39,40], ADHD [41], ID and neurocognitive impairments [42,43,44], epilepsy and seizures [17,45,46,47,48,49], global developmental delay [50], and speech and language impairments [51,52,53], alongside motor and sensory processing abnormalities [54,55,56]. In addition to these shared phenotypes, several traits display reciprocal or “mirror” effects as a function of differing gene dosage at the 16p11.2 locus. Deletions, with an assumed half-dosage or ‘haploinsufficiency’ within the deleted interval, are associated with increased body mass index and obesity. In contrast, duplications with an assumed triple dosage or ‘triplosensitivity’ are associated with reduced body mass index, reflecting dosage-sensitive regulation of metabolic pathways [36,57,58]. Similarly, reciprocal effects are observed in head size and brain structure. Deletions are typically associated with macrocephaly, increased white matter volume, reduced ventricular volume, and duplications showing the opposite pattern [50,59,60,61,62]. Importantly, this phenomenon of reciprocity is established for a limited set of macroscopic phenotypes. However, whether similar symmetry extends to underlying cellular and circuit-level mechanisms remains less clear. Despite substantial phenotypic overlap between reciprocal CNVs, important asymmetries exist. Notably, duplications of the proximal 16p11.2 locus confer a markedly increased risk for schizophrenia and psychotic disorders [25,63,64,65,66,67,68,69,70], whereas deletions do not show a comparable association.

This broad range of phenotypes associated with 16p11.2 CNVs, as well as with other recurrent CNVs linked to NDDs and NPDs, highlights two defining features of these variants: incomplete penetrance and variable expressivity [71]. Despite the presence of stereotyped breakpoints, resulting in relatively uniform size and genes impacted in the CNV, carriers of the same rearrangement on the 16p11.2 locus can significantly differ in the presence, nature, and severity of their clinical features [72]. Indeed, cohort studies of CNV carriers consistently demonstrate a more substantial variability in outcomes within CNV groups, with more limited overlap between distinct CNV loci [73], suggesting that phenotypic heterogeneity is an intrinsic property of these variants rather than a consequence of imprecise genotyping. Moreover, across many CNV loci, including 16p11.2, deletions tend to be associated with higher average penetrance and more consistent neurodevelopmental impairment, whereas duplications tend to exhibit broader phenotypic variability [74]. Duplication carriers may be mildly affected or apparently unaffected in the general population (presumably identified via an incidental finding). However, a subset exhibit severe neuropsychiatric outcomes, including frank psychotic illness, which contributes to a wider overall spectrum of expressivity [41]. Importantly, estimates of penetrance vary substantially across studies and are influenced by ascertainment bias. Genotype-first cohorts or clinically ascertained cohorts report high rates of psychiatric morbidity among duplication carriers [43,47]. This may reflect an enrichment of individuals who engage with health services. In contrast, population-based and inheritance data indicate that duplications are more frequently transmitted and have lower de novo rates than deletions (16–29% for deletions, 60–76% duplications [37,75]), consistent with a reduced average impact on reproductive fitness despite significant risk in a subset of carriers.

Phenotypic expression of 16p11.2 CNVs is clearly therefore further shaped by modifying factors beyond the primary copy number change. Secondary ‘background’ genetic variants, perinatal complications, and broader environmental influences have all been shown to modulate cognitive and behavioral outcomes in CNV carriers [6,76]. Sex also plays a significant role, with female carriers displaying relative protection from severe neurodevelopmental and psychiatric phenotypes across multiple CNV loci, including 16p11.2 [33,76,77]. In deletion carriers, CNVs may additionally unmask pathogenic recessive variants on the intact homologous chromosome, further contributing to clinical variability [78,79,80]. Hence, these observations argue against a simple single-gene model of pathogenicity for 16p11.2 CNVs. Experimental studies across multiple systems, including *Drosophila*, zebrafish, and mouse models, show that altered dosage of individual genes within the locus produces overlapping but partial phenotypes. In contrast, combined perturbation of multiple genes more closely recapitulates features of the full CNV [81,82,83,84]. Consistent with this body of evidence, theoretical frameworks propose that CNV-associated phenotypes arise through cis-epistatic interactions in which imbalanced dosages of multiple genes within the locus influence each other through shared molecular and developmental pathways to shape neurodevelopmental outcomes [74,85].

While such genetic frameworks have been essential for understanding the architecture of 16p11.2 CNVs, they do not in themselves specify the cellular or circuit-level processes through which altered gene dosage produces disease-relevant phenotypes. Single-gene models cannot fully capture the coordinated effects of simultaneous dosage perturbation across development. Here, in this review of the field, we endorse a model in which 16p11.2 CNVs disrupt neurodevelopment not through isolated molecular defects, but by altering the timing, coordination, and stabilization of synapses, with downstream effects on neuronal circuit maturation and plasticity. Accordingly, this review aims to integrate evidence from whole-locus human and animal studies with mechanistic insights from gene-specific investigations to develop a circuit-level framework for 16p11.2 pathology. Focusing sequentially on chromatin regulation and early neuronal development, followed by circuit refinement processes such as synaptic pruning and interneuron maturation, and finally on intracellular signaling mechanisms that govern activity-dependent plasticity, we examine how convergent and divergent mechanisms across reciprocal CNVs give rise to shared phenotypes, dosage-sensitive mirror traits, and asymmetries in psychiatric risk. This review is intended as a narrative synthesis of the field rather than a systematic or exhaustive survey. The studies discussed were selected based on their relevance to the key themes of CNVs, the 16p11.2 locus, neurodevelopment, and others, with the aim of highlighting representative and mechanistically informative findings. Throughout this review, we highlight both convergent and divergent effects of deletions and duplications, noting that evidence is often stronger for deletions and more limited and heterogeneous for duplications. This review is intended as a narrative synthesis of the field rather than a systematic or exhaustive survey. The studies discussed were selected based on their relevance to the key themes of CNVs, the 16p11.2 locus, neurodevelopment, and others, with the aim of highlighting representative and mechanistically informative findings.

## 2. Chromatin and Transcriptional Regulation of Neurodevelopmental Programs

A contributing factor to NDDs and NPDs is the disruption of chromatin architecture [86]. Chromatin is a macromolecular complex composed of DNA and associated proteins that plays a role in the regulation of DNA transcription and replication, as well as genomic stability [87]. Rather than constituting a separate developmental stage, chromatin regulation operates across the life course to constrain and direct both early developmental programs and later activity-dependent synaptic refinement. Precise spatiotemporal regulation of chromatin ensures that gene expression programs are activated in the appropriate cellular contexts and developmental windows [88]. In this context, Blumenthal et al. [89] demonstrated altered physical interactions between the proximal and distal 16p11.2 segments, as well as between proximal 16p11.2 and other genomic loci. These changes were attributed to disruption of chromatin folding, potentially affecting gene expression. Similar long-range chromatin effects have been described at other pathogenic CNV loci, including 22q11.2, where chromatin reconfiguration is thought to contribute to disease risk [90].

A potential mechanistic link between 16p11.2 CNVs and chromatin dysregulation lies within genes in this locus. *INO80E* encodes a component of the ATP-dependent chromatin remodeling protein complex INO80 [91], which regulates nucleosome organization and transcription during cortical neurogenesis. The proteins in this family promote histone exchange and modulate chromatin accessibility, thus facilitating transcriptional activation necessary for the execution of key gene expression programs during development [88]. The locus also contains the gene *HIRIP3*, which encodes for a histone-interacting protein involved in histone deposition and chromatin assembly [92]. These two genes are arranged in a head-to-head orientation [93], although the functional consequences of this genomic organization remain poorly understood. Transcriptomic studies have implicated altered *INO80E* expression in schizophrenia [94]. Moreover, overexpression of *INO80E* in the medial prefrontal cortex of wild-type mice has been shown to induce cellular and behavioral phenotypes similar to those observed in schizophrenia [95], including altered neuritogenesis and dysregulation of genes linked to synaptic and neuronal function. In carriers of 16p11.2 duplication, which is a known risk factor for schizophrenia, *INO80E* expression is increased in a gene dosage-dependent manner [89].

These findings raise the possibility that maladaptive chromatin remodeling may contribute to psychiatric vulnerability associated with the duplication, though further research will be necessary to establish a causal relationship. Importantly, chromatin-mediated dysregulation remains modifiable. Using a mouse model of the 16p11.2 duplication, Rein et al. [96] reported increased expression of histone deacetylase (HDAC5) and reduced histone acetylation at promoters of activity-dependent genes. Histone acetylation plays a key role in gene transcription by weakening histone–DNA interactions and locally decompacting chromatin [97]. Elevated HDAC5 hence results in an inhibition of gene transcription, which manifested in the 16p11.2 duplication mouse model as impaired GABAergic synaptic transmission, and was rescued through pharmacological inhibition of HDAC5 [96]. This illustrates how epigenetic dysregulation constitutes a functionally relevant and potentially reversible layer of pathology, linking altered gene dosage to downstream synaptic impairments.

## 3. Early Developmental Perturbations That Set Synaptic Trajectories

Early brain development is governed by tightly coordinated processes, including neural progenitor proliferation, migration, and differentiation, which are regulated by precisely timed gene expression programs and signaling pathways [98]. Although synaptogenesis and synaptic pruning occur at later developmental stages, early events determine key neuronal properties, including cell identity, positioning, and intrinsic connectivity potential. Thus, early developmental processes establish biological scaffolds that later shape synapse formation and refinement. Disruptions of early developmental programs are central to the etiology of many NDDs and NPDs [99].

In the context of recurrent CNVs such as 16p11.2, altered gene dosage has the potential to perturb developmental trajectories prior to synapse formation, thus biasing downstream circuit assembly and maturation. Advances in human stem-cell-derived model systems have enabled direct investigation of these early processes in a human genetic context [100]. Using induced pluripotent stem-cell-derived cortical organoids from 16p11.2 CNVs, Urresti et al. [101] demonstrated that both deletions and duplications are associated with impairments in neuronal migration and delayed maturation. In deletion organoids, these changes were accompanied by an excess of neurons, likely driven by elongation of the neuronal cell cycle, which promotes early maturation and premature depletion of the neural progenitor pool. In contrast, duplication organoids did not show a comparable change in number of neurons. Moreover, the authors reported gene dosage-dependent effects on neurite growth and synaptic markers. Deletion organoids showed an increased neurite length and synaptic puncta density, whereas duplication organoids showed the opposite trend. Another study on cerebral organoids by Tai et al. [102] reported evidence for differences in ratios of excitatory and inhibitory neurons in 16p11.2 CNV models. These studies support the conclusion that gene copy number at this locus strongly influences early neuronal migration and maturation. A potential contributor to these effects is the *QPRT* gene within the locus. This gene encodes for quinolinate phosphoribosyltransferase, an enzyme involved in NAD+ biosynthesis [103]. Beyond this role, *QPRT* is implicated in neuronal development; blocking of *QPRT* function in neuronal precursor cultures results in impaired cell survival, neuronal differentiation, and neurite complexity, suggesting a role in the regulation of the cytoskeleton [104]. Hence, evidence suggests that altered gene dosage at the 16p11.2 locus has direct consequences for early neuronal developmental processes. By disrupting the timing and progression of these key events, these CNVs have an impact on the number, identity, and position of cortical neurons that participate in synapse formation.

## 4. Synaptic Formation and Refinement

Following neuronal migration and differentiation, developing circuits undergo a prolonged period of synaptic formation and refinement that extends from the late prenatal stages through to postnatal development and adolescence (reviewed in [105]). During this phase, the brain initially generates an excess of synaptic connections. These are subsequently eliminated through activity-dependent synaptic pruning, a process essential for efficient circuit formation. Synaptic pruning is primarily mediated by microglia, which selectively engulf weak or inactive synapses while sparing functionally relevant ones [106]. This selective elimination depends on a tightly regulated balance between pro- and anti-phagocytic signals [107]. Synapses destined for removal are tagged by components of the classical complement cascade, most notably C1q and C3, which act as “eat me” signals recognized by complement receptors expressed on microglia [108]. In contrast, active synapses are protected by counteracting “don’t eat me” signals, ensuring that synaptic pruning remains spatially and temporally specific [109]. Disruption of this balance leads to abnormal synapse numbers and altered circuit organization, and aberrant synaptic pruning has been implicated across a range of neurodevelopmental and neuropsychiatric disorders (reviewed in [110]).

Recent evidence indicates that synaptic pruning is directly disrupted in carriers of 16p11.2 copy number variants [111], with converging data pointing to altered regulation of complement mediated and anti-phagocytic signaling. A key locus gene in this context is *SEZ6L2*, a member of the seizure-related 6 (SEZ6) protein family. SEZ6 proteins are localized to synapses and have been implicated in dendritic development and synaptic plasticity [112]. Notably, several SEZ6 family members act as inhibitors of complement-mediated synapse opsonization, limiting C1q and C3 deposition and thereby protecting synapses from microglial engulfment. Among these, SEZ6L2 protein exhibits particularly strong inhibitory activity. Loss of SEZ6L2 results in impaired excitatory synaptic connectivity, consistent with a role in fine-tuning synaptic refinement [113]. In parallel, dysregulation of anti-phagocytic signaling has emerged as another mechanism contributing to impaired pruning in 16p11.2 deletion models. The transmembrane protein CD47 functions as a “don’t eat me” signal by engaging the inhibitory receptor SIRPα on microglia and suppressing phagocytosis [109]. CD47 expression is significantly upregulated in human cellular models derived from 16p11.2 deletion carriers, leading to reduced microglial synaptic engulfment [114]. Notably, these cells also show increased surface expression of calreticulin, a pro-phagocytic cue, suggesting that excessive CD47 signaling can override elimination signals that would normally promote synaptic pruning. These findings have been replicated in vivo; mouse models of 16p11.2 deletion show reduced microglial phagocytic activity, increased density of excitatory synapses, and enhanced synaptic transmission [115]. Pharmacological blockade of CD47 restores microglial engulfment and rescues synaptic phenotypes, supporting a causal link between dysregulated CD47 signaling and impaired synaptic refinement. Complement-based mechanisms may further contribute to deletion-associated phenotypes. Increased C3 expression and inflammatory marker levels have been observed in deletion models, and inhibition of downstream complement signaling alleviates behavioral abnormalities [111].

Together, these results indicate that 16p11.2 deletions disrupt synaptic pruning by shifting the balance of pruning signals toward synapse retention. This provides a plausible mechanistic explanation for deletion-associated features such as increased synaptic density, network hyperconnectivity, and increased brain volume. In contrast, evidence addressing corresponding mechanisms in duplication models remains more limited. Clinically, 16p11.2 duplications are associated with reduced head size and brain volume, suggesting the possibility of reciprocal neurodevelopmental effects. One potential, though yet unproved, explanation is that increased dosage could lead to relatively enhanced or accelerated synaptic pruning. While complement-mediated synaptic elimination is a possible pathway, direct evidence linking this mechanism to duplication-associated phenotypes is currently lacking. Overall, these findings position synaptic pruning as a critical, dosage-sensitive vulnerability point within the 16p11.2 locus.

## 5. Interneuron Maturation, Perineuronal Nets, and Critical Period Regulation

For functional circuits to emerge, the highly plastic period of postnatal synaptic refinement must eventually transition toward stability. While synaptic pruning reduces excess connectivity, circuit maturation also requires the closure of developmental critical periods, enabling reliable network dynamics and information processing [116]. Disruption of the timing of this transition, whether through premature or delayed stabilization, can have lasting consequences for circuit function. This transition is tightly regulated by interneurons, particularly by parvalbumin-positive (PV+) GABAergic interneurons, which play central roles in maintaining local circuit excitation/inhibition (E/I) balance and generating gamma-frequency network oscillations essential for cognition [117]. Maturation of PV+ interneurons is coupled with the formation of perineuronal nets (PNNs), specialized extracellular matrix assemblies that enwrap the soma and proximal neurites of these cells [118]. As PNNs mature, they regulate PV+ interneuron excitability and physically stabilize existing synaptic contacts, thereby restricting further activity-dependent remodeling. Therefore, PNN formation timestamps the functional closure of the critical period of heightened plasticity. This is confirmed by studies in which enzymatic degradation of PNNs partially reactivates juvenile-like synaptic plasticity [119,120], framing PNNs as regulators of circuit stability rather than passive structural components.

This stabilization process is disrupted in 16p11.2 CNVs. Willis et al. [121] reported that in mouse models of 16p11.2 duplication, BDNF-responsive signaling and JNK pathway activity are upregulated, which is accompanied by accelerated maturation of PV+ interneurons and premature formation of PNNs. The consequences of these disruptions persisted into adulthood, with increased PNN density and dysregulated inhibitory markers in the prefrontal cortex and thalamic reticular nucleus, consistent with early critical period closure and aberrant circuit stabilization, leading to pathological disinhibition. However, it should be noted that early acceleration of interneuron development is not specific to duplications at 16p11.2. Human ventral telencephalic organoid models of the reciprocal deletion also exhibit premature interneuron differentiation [122], which suggests that altered timing of interneuron maturation may be a shared consequence of changes in 16p11.2 dosage. In deletion models, this early maturation is followed by reduced expression of interneuron markers and impaired functional connectivity in later developmental stages [123]. Although PNN development itself has not yet been assessed in deletion models, this evidence raises the possibility that PV formation and PNN maturation may be tightly coupled processes that are disrupted in timing rather than direction across reciprocal CNVs. Rather than producing mirror phenotypes, 16p11.2 deletions and duplications may therefore converge on a shared vulnerability in the coordination of plasticity and stability. Early or mistimed engagement of PV-PNN-dependent mechanisms could lead to insufficient refinement in some circuits or excessive stabilization in others.

## 6. Mapping Networks of Signaling Pathways That Converge on Synaptic Function

Rather than defining discrete developmental stages, intracellular and extracellular signaling pathways operate as continuous modulators of neuronal and synaptic function across development. These pathways shape neural circuits by regulating the timing, magnitude, and stability of developmental processes. Converging genomic evidence indicates that many risk genes implicated in NDDs act both during early development and in regulating synaptic plasticity in the mature brain. This suggests that altered plasticity may represent a persistent vulnerability rather than a time-limited effect [124]. Alterations in gene dosage at loci such as 16p11.2 CNV have the potential to perturb signaling balance over extended windows, biasing synaptic and circuit function at multiple levels.

Studies examining the impact of 16p11.2 CNV on circuit properties using data from 16p11.2 carriers, as well as rodent, organoid, and iPSC-derived cell culture models, have identified robust circuit-level abnormalities across model systems and experimental modalities. These include disruptions in neuronal oscillations [102,125,126,127], functional network connectivity [123,128,129], synaptic plasticity and function [130,131,132,133,134], and dysregulated E/I balance [135]. Although phenotypes vary across brain regions and models, synaptic and circuit dysfunction is a consistent feature, which suggests convergence at the level of intracellular signaling mechanisms. This section focuses on signaling pathways through which altered 16p11.2 gene dosage biases synapse and circuit development across time.

### 6.1. Key Hub Signaling Pathways

#### 6.1.1. ERK/MAPK

ERK/MAPK pathways are ubiquitous regulatory cascades that respond to diverse extracellular cues, such as hormones, neurotransmitters, and growth factors [136,137], and play critical roles in neuronal survival, morphological maturation, cell proliferation, and synaptic plasticity [138,139,140]. In neurons, ERK activity is tightly coupled to intracellular calcium dynamics, enabling neuronal activity to influence transcriptional programs and structural plasticity [141]. The 16p11.2 locus contains the *MAPK3* gene encoding for the ERK1 protein, a core component of the ERK1/2 cascade [142]. Network-level analyses have identified the ERK/MAPK pathway as a highly connected hub within the protein–protein interaction networks of the 16p11.2 locus [143], suggesting that even modest changes in ERK signaling could exert widespread downstream effects. ERK1/2 protein activity integrates signals across developmental and synaptic contexts [144], positioning *MAPK3* as a key mediator through which altered gene copy number may influence circuit organization. Experimental evidence supports a dose- and timing-sensitive role for ERK in brain development [81]. Disruption of ERK signaling during cortical development alters neural progenitor dynamics, neuronal excitability, and behavior [145], whereas postdevelopmental ERK dysregulation impairs dendritic architecture [146], synaptic plasticity [147], and experience-dependent circuit function [144].

In mouse models of 16p11.2 deletion, ERK1/2 activity is increased, along with alterations in neuronal morphology and network function [148]. Moreover, peripheral ERK1 protein levels mirror *MAPK3* gene dosage in individuals with 16p11.2 CNVs, with reduced levels in deletion carriers and increased levels in duplication carriers [149]. Importantly, pharmacological modulation of the Ras-ERK pathway in mice has been shown to rescue aspects of synaptic and behavioral dysfunction, particularly when intervention is timed to early developmental windows [150,151]. Conversely, duplications of the 16p11.2 locus may potentially be associated with reduced or dysregulated ERK output, potentially contributing to distinct phenotypic outcomes between reciprocal CNVs. Across species, perturbation of MAPK/ERK pathway disrupts axon targeting, synaptic specificity and circuit wiring [81,152,153], supporting a role for ERK signaling in establishing and maintaining precise neural connectivity. Altered *MAPK3* copy number biases ERK levels, leading to miscoordination of synaptic plasticity across individual synapses, as well as across distributed neural circuits. Circuits undergoing prolonged postnatal maturation and experience-dependent refinement, such as corticocortical, thalamocortical, and hippocampal networks [154,155], may be particularly vulnerable to shifts in ERK dosage.

#### 6.1.2. KCTD13-Cul3-RhoA

RhoA is a small Rho-family GTPase that regulates actin cytoskeleton dynamics and thus influences cell morphology, migration, and synaptic structure [156]. In excitatory neurons, particularly pyramidal neurons, RhoA localizes to cell bodies and dendrites, playing roles in shaping dendritic architecture and spine organization [157]. Elevated postsynaptic RhoA activity is associated with reduced dendritic complexity and impaired synaptic transmission without impacting intrinsic membrane excitability, highlighting the role of this molecule as a structural rather than electrophysiological modulator [158]. RhoA levels are tightly regulated by ubiquitin-mediated protein degradation [159]. Within the 16p11.2 locus, the *KCTD13* gene encodes for an adaptor protein for the Cullin-3 (Cul3) E3 ubiquitin ligase complex [160,161], targeting RhoA for ubiquitination and subsequent degradation. Through this mechanism, it acts as a brake on RhoA signaling in postmitotic neurons [162]. As such, the KCTD13-Cul3-RhoA axis may represent a dosage-sensitive signaling pathway through which altered gene copy number can bias neurite dynamics, cytoskeletal stability, and synaptic function [163].

Experimental manipulation of *KCTD13* dosage produces phenotypes that recapitulate certain features of 16p11.2 CNV syndromes, although incompletely [82,164]. Reduction in *KCTD13* expression in mouse and cell models leads to increased RhoA activity [164], neurite outgrowth, reduced synaptic transmission [165], and deficits in hippocampal-dependent learning and memory [166]. Importantly, pharmacological inhibition of RhoA or its downstream effectors, including ROCK kinases, has been shown to rescue multiple synaptic and behavioral phenotypes, supporting a possible causal role for excessive RhoA signaling in 16p11.2 deletion-associated dysfunction [167,168]. However, the relationship between 16p11.2 dosage and RhoA signaling may not be strictly linear; both deletions and duplications of the 1611.2 locus have been reported to result in RhoA overactivation in organoid models, stalling neuronal migration and altering circuit assembly [101]. These findings indicate that RhoA dysregulation arises not solely from loss of *KCTD13* function, but from broader perturbation of interacting pathways across the locus. Indeed, differences in phenotypes observed across various *KCTD13* loss-of-function models, ranging from full gene deletion [164] to exon-specific knockouts [82,165,166] suggests that *KCTD13*-mediated effects interact epistatically with other 16p11.2 genes rather than operating as a standalone driver. Beyond RhoA regulation, *KCTD13* has been implicated in additional synaptic mechanisms. For example, evidence suggests that this protein mediates the ubiquitination and degradation of the NMDA receptor subunit GluN1, thus influencing glutamate receptor surface expression and excitatory synaptic transmission [161]. This reinforces the view that *KCTD13* contributes to synapse regulation via multiple routes. Together, these studies suggest that the KCTD13-Cul3-RhoA pathway is a mediator through which 16p11.2 gene dosage may disrupt synaptic stability.

#### 6.1.3. Npas4

Npas4 is an activity-dependent immediate early gene that plays central roles in the regulation of circuit homeostasis and synaptic plasticity [169]. Npas4 expression is rapidly induced by postsynaptic calcium influx following neuronal activity. It then regulates downstream genes, including ion channels, signaling molecules, and other transcription factors, thus influencing synapse formation, maintenance, and function [170]. This is executed primarily via coordination of inhibitory synapse development, wherein Npas4 promotes the formation and stabilization of inhibitory input onto excitatory neurons and excitatory input onto inhibitory neurons [171,172]. As such, Npas4 maintains local E/I balance. Through this mechanism, the gene contributes to experience-dependent plasticity, learning and memory, as well as the long-term stability of neural networks. Loss of Npas4 disrupts these processes and has been associated with impairments in social behavior, cognition, and learning [173]. In the context of 16p11.2 CNVs, Npas4 has emerged as a downstream node linking altered signaling dynamics to inhibitory circuit dysfunction. In a mouse model of the 16p11.2 duplication, Npas4 expression was found to be downregulated in the prefrontal cortex, manifesting as impaired GABAergic synaptic transmission, as well as social and cognitive deficits [174]. Targeted restoration of Npas4 expression in the prefrontal cortex was sufficient to rescue these impairments. This indicates that Npas4 dysregulation contributes directly to circuit-level pathology in this model.

### 6.2. Locus Genes Shaping Signaling Dynamics

Rather than acting as independent effects, many genes within the 16p11.2 locus converge on a number of signaling functions that regulate how neuronal activity is translated into synaptic and circuit-level change. These functions include (i) modulation of activity-dependent signaling cascades, (ii) regulation of neurotransmitter release dynamics, and (iii) control of cytoskeletal and structural plasticity. The genes discussed below illustrate how dispersed locus components functionally interact within these shared pathways. Other genes within the locus may contribute to these mechanisms and are likely to interact with them in a context-dependent manner.

MVP (major vault protein) is the principal structural component of vault ribonucleoprotein particles and has been implicated in nucleocytoplasmic transport during brain development [81]. In cortical postmitotic neurons, MVP participates in the transport of mRNAs along neurites, supporting local protein synthesis in synapses [175]. MVP has also been shown to interact with and amplify ERK signaling at synaptic sites [176], suggesting a role as a modulator of activity-dependent signaling cascades. Via these interactions, MVP contributes to the regulation of neuronal morphology [177] and experience-dependent cortical plasticity [178], suggesting that altered *MVP* gene dosage may influence synaptic responsiveness by shaping local translation and ERK-dependent plasticity. Thus, *MVP* links activity-dependent signaling cascades to synaptic protein synthesis, acting as a modulator of downstream plasticity mechanisms described in earlier sections.

At the presynaptic level, *PRRT2* and *DOC2A* represent complementary regulators of neurotransmitter release dynamics. PRRT2 (proline-rich transmembrane protein 2) is a presynaptic membrane protein that interacts with fast calcium sensors, such as synaptotagmin-1/2 and components of the SNARE complex, thus influencing evoked vesicle release probability [179,180]. Loss-of-function mutations in the *PRRT2* gene lead to synaptic transmission deficits and are strongly associated with convulsive disorders, such as paroxysmal kinesigenic dyskinesia and infantile epilepsy [181,182]. Notably, carriers of the 16p11.2 duplication also show increased susceptibility to paroxysmal movement disorders and seizures, raising the possibility that *PRRT2* overexpression contributes to circuit hypersynchrony and seizure liability [183,184]. How *PRRT2* dosage interacts with other locus genes to shape this phenotype is currently unknown; nonetheless, these observations highlight dosage sensitivity at the level of presynaptic release machinery.

In contrast, DOC2A (double C2 domain alpha) protein functions as a calcium sensor involved in slow asynchronous and spontaneous glutamate release with minimal impact on fast action potential-evoked transmission [185]. *DOC2A* knockout reduces spontaneous glutamate release and dampens hippocampal activity [186,187]. Given the established role of spontaneous neurotransmission in homeostatic plasticity and long-term regulation of synaptic strength, DOC2A is thought to influence circuit tuning indirectly. It shapes baseline synaptic and network states rather than directly mediating rapid transmission or long-term potentiation [188]. DOC2A has also been linked to activity-dependent transcriptional programs, including the plasticity-associated transcription factor Npas4 described above [189]. Duplications of *DOC2A* are associated with increased schizophrenia risk, suggesting that this may lead to excessive spontaneous synaptic activity or altered calcium-dependent signaling, destabilizing network coordination [190]. Moreover, the gene was found to interact with the lipid-metabolism gene *FAM57BA* on the same locus, wherein combined disruption of these genes resulted in hyperactivity and seizure susceptibility [191], thus underlining cross-pathway epistasis within the 16p11.2 locus. Together, *PRRT2* and *DOC2A* highlight how 16p11.2 genes can influence complementary aspects of synaptic transmission, from fast-evoked to slower spontaneous vesicle release. These processes are closely linked to activity-dependent signaling pathways, including those involving Npas4, suggesting convergence between presynaptic release dynamics and transcriptional regulation of circuit homeostasis.

Finally, *TAOK2* (thousand-and-one amino acid kinase 2) emerges as an integrator of extracellular signals to cytoskeleton organization and synaptic structure. TAOK2 is a serine–threonine kinase involved in processes such as neuronal migration, dendritic arborization, protein translation, and synaptic connectivity [192,193,194]. This protein regulates septin, actin, and microtubule function [195] via stress-activated MAPK pathways, including JNK and p38 [196], as well as through crosstalk with RhoA signaling [197]. Experimental evidence demonstrates that knockdown of *TAOK2* leads to delayed neuronal migration [198] and deficits in dendritic arborization and branching [192,197,199] due to destabilization of cytoskeletal components. In cortical excitatory neurons, loss of TAOK2 disrupts synaptic density and connectivity and induces behavioral abnormalities [200]. In contrast, overexpression of *TAOK2* significantly increases dendritic complexity, drives excessive cytoskeleton stabilization and pushes premature cell maturation, leading to a decrease in neurite plasticity [199]. Importantly, TAOK2 interfaces with both MAPK and RhoA pathways [151,197], positioning it as a point of convergence between the signaling cascades and structural plasticity mechanisms described earlier. Through these interactions, TAOK2 may coordinate how extracellular signals are translated into lasting changes in neuronal architecture.

These genes do not define isolated molecular mechanisms; instead, they converge on the conversion of neuronal activity into stable synaptic and circuit-level changes. Across the examples discussed above, a theme emerges in which 16p11.2 gene dosage perturbs the balance between signaling input, synaptic transmission, and structural plasticity. Disruption of this balance is likely to have cumulative effects across development, contributing to the circuit-level phenotypes described in earlier sections.

### 6.3. Subcellular Sites of Signaling Convergence

The signaling pathways and locus genes described in the above sections converge on discrete subcellular compartments that spatially constrain, integrate, and amplify synaptic signaling. Rather than acting diffusely throughout the neuron, these pathways are organized into specialized domains that shape how extracellular cues are translated into intracellular responses. Two such compartments, neuronal primary cilia and dendritic spines, have emerged as key sites at which 16p11.2 gene dosage influences synaptic plasticity.

#### 6.3.1. Primary Cilia

Primary cilia are microtubule-based membrane protrusions present in most mammalian cell types, which have been long recognized for their roles in early brain development [201,202]. Although initially considered vestigial in mature neurons, it is now clear that primary cilia persist in postmitotic neurons and retain important cellular functions [203]. In mature neurons, primary cilia act as specialized signaling compartments that concentrate G-protein-coupled receptors, ion channels, and second messenger machinery. This enables them to respond to extracellular cues and modulate intracellular signaling cascades with high spatial specificity. Recent work has demonstrated that neuronal primary cilia actively participate in synaptic signaling, including the formation of direct axon–cilium synaptic contacts [204,205,206]. Moreover, they contribute to the integration of neurons into functional circuits following migration [203] and regulate homeostatic synaptic plasticity by dynamically adjusting their length in response to network activity [207]. Consistent with these roles, ciliary signaling has been implicated in learning and memory processes as well as the maintenance of network excitability.

Disruption of primary cilia has profound consequences for brain function and circuit organization [202]. Impaired ciliogenesis or ciliary signaling alters the development and connectivity of GABAergic interneurons [208], leading to enhanced excitatory drive and perturbations in excitatory/inhibitory balance at the network level [209]. Such mechanisms have been proposed to contribute to the pathophysiology of neurodevelopmental and neuropsychiatric disorders, including autism spectrum disorder, schizophrenia, and bipolar disorder [210,211,212]. Emerging evidence suggests that primary cilia is also affected in the context of 16p11.2 CNV. In a mouse model of the 16p11.2 duplication, Migliavacca et al. [213] reported structural abnormalities of primary cilia in hippocampal CA1 neurons. Complementing these findings, Byeon et al. [214] demonstrated gene dosage-dependent alterations in ciliary morphology in iPSC-derived neural progenitor cells, with elongated primary cilia observed in deletions and shortened cilia in duplications. Manipulation of *TAOK2* expression was sufficient to recapitulate these effects, implicating locus-encoded signaling regulators as contributors to ciliary dysregulation in 16p11.2 CNVs. Notably, the involvement of TAOK2 kinase in regulating ciliary length is consistent with its established role in coordinating cytoskeletal stability and signaling dynamics, suggesting that altered *TAOK2* gene dosage may influence multiple subcellular compartments through shared cytoskeletal mechanisms.

#### 6.3.2. Dendritic Spines

While primary cilia integrate neuromodulatory and activity-dependent signals at the level of the soma and nucleus, many of the downstream effects of these pathways are ultimately realized at excitatory synapses. Dendritic spines spatially constrain signaling at individual synapses, allowing fine grained regulation of synaptic strength and plasticity [215]. Structurally, spines are small actin-rich protrusions composed of a bulbous head connected to the dendritic shaft by a narrow neck, which biochemically and electrically isolates synaptic signaling from the dendrite [216]. Dendritic spines can be broadly classified into thin, stubby, and mushroom subtypes, reflecting differences in morphology and stability [217]. Thin spines are immature and highly plastic with a role in learning, stubby spines are common during development, and mushroom spines are involved in mature and stable synapses. Spine stability and maturation are tightly coupled to synaptic function and depend on coordinated regulation of actin cytoskeleton and postsynaptic scaffolding complexes [216].

Dendritic spine dysfunction has been implicated in the etiology of multiple neurodevelopmental and neuropsychiatric conditions, with observations of disease-specific disruptions in their size, shape, or number [218]. In this context, some 16p11.2 locus genes implicated in intracellular signaling have been shown to influence dendritic spine morphology and maturation. Notably, *TAOK2* contributes to spine maturation through phosphorylation of cytoskeleton component septin-7, which stabilizes the postsynaptic density scaffolding protein PSD-95 and promotes synaptic stability [219]. In parallel, deletion of the *KCTD13* gene was found to result in reduced spine density and impaired spine maturation in hippocampal CA1 pyramidal neurons [164,166]. Although TAOK2 has been shown to interact with RhoA, a downstream target of *KCTD13* activity, evidence suggests that spine maturation deficits associated with *KCTD13* occur independently of RhoA signaling [166], highlighting mechanistic heterogeneity within the 16p11.2 pathway. Consistent with these findings, neurons derived from individuals with the 16p11.2 duplication exhibit excessive dendritic branching and increased spine numbers. These changes are accompanied by abnormal AMPA receptor expression and impaired homeostatic synaptic scaling, indicating disrupted neuronal plasticity [220]. These findings indicate a failure to properly stabilize synaptic strength in response to network activity, further reinforcing the idea that 16p11.2 gene dosage disrupts not only synapse number, but also the rules governing synaptic refinement and plasticity.

## 7. An Integrated Framework for Translating Psychiatric Risk in 16p11.2 CNVs

**Figure 1:**
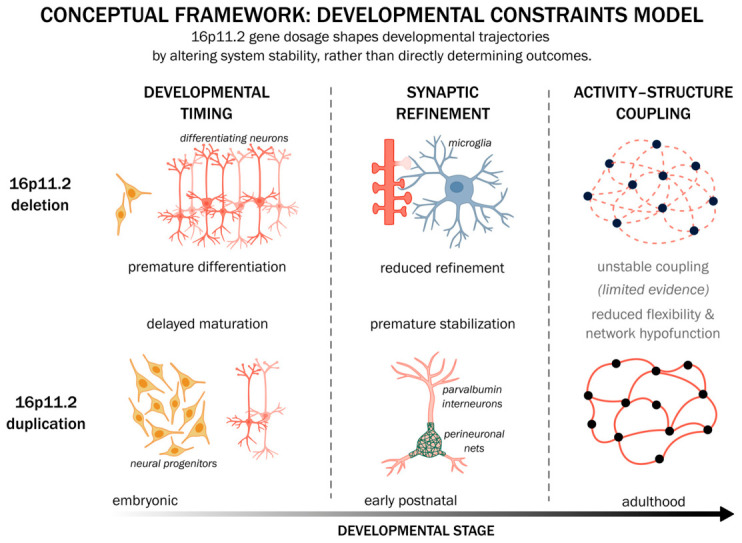
Conceptual framework illustrating a three-stage developmental constraint model linking 16p11.2 copy number variation to circuit-level phenotypes. The horizontal axis denotes developmental progression from embryonic stages through early postnatal circuit formation to adult network function. Three sequential and partially overlapping domains are illustrated: (1) developmental timing, (2) synaptic refinement, and (3) activity–structure coupling. Empirical findings supporting these domains are represented by the specific processes depicted within each stage, while the organization of these processes into a sequential “constraints” framework reflects the conceptual synthesis proposed here. In early development, 16p11.2 deletions are associated with accelerated neurogenesis, including premature differentiation and depletion of neural progenitor pools, whereas duplications show delayed maturation and prolonged maintenance of progenitors. During postnatal circuit formation, both genotypes are associated with altered timing of plasticity-related processes, including maturation of parvalbumin-positive (PV) interneurons and associated perineuronal nets (PNNs). These changes are associated with reduced refinement in deletion models, whereas duplication-associated effects on synaptic stabilization remain less well characterized. At the level of mature circuit function, these developmental perturbations are associated with differences in activity–structure coupling. Deletions may be linked to less stable and poorly coordinated network activity, whereas duplications may involve increased stabilization and reduced flexibility of network dynamics, potentially consistent with reports of circuit hypofunction. This model emphasizes that reciprocal 16p11.2 CNVs do not produce simple mirror phenotypes but instead alter the temporal coordination of developmental processes, thereby shaping system-level vulnerability to divergent circuit outcomes. Mechanistic contributors are not exhaustively depicted but are discussed in the main text. Arrow directions and panel organization reflect inferred relations and should be interpreted as conceptual rather than strictly causal.

The preceding sections describe a wide range of genes, signaling pathways, and cellular processes affected by copy number variation at the 16p11.2 locus. A straightforward but rather misleading interpretation would be to aggregate these findings into a single, cumulative pathogenic model, implying that simultaneous disruption of multiple mechanisms inevitably produces severe neurodevelopmental dysfunction. However, such a simplistic approach would be inconsistent with the clinical reality of the 16p11.2 carrier population, characterized by the marked phenotypic variability and incomplete penetrance, as described in the Introduction. Rather than viewing the effects of 16p11.2 dosage imbalance as additive deficits, we argue that they are better understood as developmental constraints that bias how neural circuits form, adapt and stabilize over time. In this context, “constraints” do not imply deterministic limitations. Instead, they describe biases in developmental trajectories that increase sensitivity to subsequent perturbations. Importantly, the framework illustrated in Figure 1 is intended as a conceptual model rather than a definitive causal account. While a growing body of evidence links 16p11.2 dosage to alterations in molecular, cellular, and circuit-level processes, much of this work derives from model systems or isolated experimental contexts. Accordingly, the mechanisms discussed below are best interpreted as sources of increased developmental vulnerability. Whether these constraints manifest as neurodevelopmental or neuropsychiatric phenotypes ultimately depends on their interaction with additional genetic, environmental, and developmental factors [221]. Across the evidence reviewed in this article, disruptions associated with 16p11.2 CNVs suggest convergence across three partially overlapping domains that link molecular and cellular alterations to circuit-level outcomes. These domains provide a theoretical framework for understanding how distributed molecular perturbations may scale into circuit-level instability and behavioral risk. We hope that this framework will help to inform the design of future preclinical and clinical research.

### 7.1. Developmental Tempo and Early Fate Commitment

Several lines of evidence indicate that 16p11.2 CNVs bias early neurodevelopmental dynamics, exerting an effect on the timing of progenitor proliferation, differentiation, and neuronal migration [101]. Multiple genes in this locus either directly encode for or interact with chromatin regulators and signaling molecules, including *INO80E*, *QPRT*, *MAPK3*, and *KCTD13* [95,104,145,163], all of which play established roles in early cortical development. Human organoid and animal models frequently report altered progenitor dynamics, impaired migration, precocious maturation, and changes in neuronal compositions for both deletions and duplications [101,102,122]. Importantly, these effects appear to reflect altered tempo and commitment, rather than failure of the developmental programs themselves. These changes in the number, position, and function of excitatory and inhibitory neurons are likely to influence downstream circuit assembly and function [116]. Early dysregulation of neuronal migration, cortical layering and early differentiation are well-documented neurodevelopmental disorders, such as macrocephalic ASD [222,223], SCZ [224], and others outside the context of 16p11.2 CNVs, supporting the scenario that early biases introduced by altered 16p11.2 gene dosage increase vulnerability to later circuit-level dysfunction. Importantly, such early alterations are unlikely to determine specific phenotypic outcomes in isolation but may instead bias the developmental landscape in ways that increase susceptibility to subsequent perturbations affecting circuit maturation.

### 7.2. Synaptic Plasticity Allocation and Stabilization Timing

A second major axis of convergence likely lies in processes that manage synaptic formation and refinement, as well as the transition from plastic to stabilized circuits. Evidence reviewed in earlier sections has indeed demonstrated that 16p11.2 dosage disrupts microglia-mediated synaptic pruning, interneuron maturation, and perineuronal net development. These are key mechanisms that determine when and how synaptic plasticity is restricted. Disruptions at this level are expected to alter how synaptic connections are selectively retained or eliminated, thereby shaping network architecture and functional connectivity.

In deletion models, some studies suggest that changes in regulation of complement mediated pruning combined with elevated anti-phagocytic signaling may favor synapse retention [114,115], consistent with increased synaptic density and macrocephaly observed in a subset of carriers [62]. In duplication models, the observed accelerated maturation of PV+ interneurons and premature perineuronal net formation are consistent with the scenario of an earlier closure of critical periods and excessive stabilization [121], although direct evidence for pruning-related mechanisms in duplication models remains limited. Although direct evidence is asymmetrical between reciprocal CNVs, these patterns align with broader hypotheses implicating similar disruptions in the etiology of NDDs and NPDs. Schizophrenia is associated with the synaptic hypothesis, which points towards excessive or mistimed synaptic pruning during highly plastic neurodevelopmental periods as key factors underlying the etiology of this disorder [225,226]. In contrast, although ASD is not directly linked with synaptic pruning or microglial function [227], studies have implicated excessive synaptogenesis and delayed network stabilization [223,228]. Taken together, these observations raise the possibility that disruptions in these processes observed in models of 16p11.2 CNVs represent a significant developmental constraint, increasing vulnerability towards the manifestation of NDDs.

### 7.3. Activity-to-Stability Coupling in Later Development

Finally, altered 16p11.2 gene dosage is associated with changes in mechanisms that translate neuronal activity into durable circuit changes during later development and adulthood. Activity-dependent signaling pathways involving ERK/MAPK, NPAS4, RhoA, calcium-dependent synaptic release, dendritic spines, and primary cilia have been implicated across several model systems [152,163,174,183,214]. These pathways link neuronal activity to synaptic and structural plasticity, thereby influencing how transient signaling events are consolidated into stable circuit configurations. In deletions, we see evidence of reduced dendritic complexity and maturation, as well as impaired glutamatergic synaptic transmission. This may partially contribute to inefficient or erratic circuit stabilization, potentially resulting in more labile network dynamics. In contrast, the combined evidence of enhanced calcium-dependent synaptic transmission, impaired inhibitory neurotransmission, and excessive dendritic complexity in duplications may be consistent with increased or earlier network stabilization, potentially reducing the flexibility of circuit responses in the face of changing environmental or developmental demands. This is also consistent with reports of reduced network responsiveness in duplication models [134]. Outside of the 16p11.2 context, dysregulation of the ERK pathway has been reported in the brains of individuals with schizophrenia [229], and disruption in NPAS4 and RhoA function is linked to NDDs [169,230]. Hence, such differences provide a potential mechanistic bridge between early developmental constraints and later emerging vulnerabilities, including adolescence-onset psychiatric symptoms.

Together, these axes suggest a model in which 16p11.2 CNVs bias neural development by constraining developmental tempo, plasticity allocation, and activity-dependent stabilization, rather than by defining fixed pathological outcomes. In this view, the constraints do not define specific outcomes, but shape the conditions under which additional genetic, environmental, and developmental factors influence emergent phenotypes. Thus, this framework accommodates for the known manifestations of phenotypic heterogeneity and incomplete penetrance, and links molecular and cellular findings to circuit-level vulnerabilities. Furthermore, it contributes towards a model wherein neurodevelopmental outcomes emerge from complex interactions between developmental trajectories and external factors related to personal lived experiences, rather than deterministic genetic effects alone.

## 8. Future Directions

A diverse range of preclinical interventions has demonstrated partial rescue of behavioral, synaptic, or circuit phenotypes in 16p11.2 mouse models; these include the targeting of glutamatergic and GABAergic signaling, modulation of ERK/MAPK activity, correction of gene dosage, or manipulation of neuromodulatory pathways. These interventions typically rescue specific phenotypes, such as hippocampal-dependent memory or behavioral deficits, rather than producing global normalization. Although these studies support the perspective that restoring network balance and synaptic plasticity is a rational intervention strategy, these rescues are usually pathway-specific and thus limited in their capacity. Importantly, most of these approaches have been performed exclusively on rodent systems, raising translational challenges for application on human CNV carriers. Hence, they should not be interpreted as evidence that identification of shared signaling pathways directly translates into viable therapeutic targets. In addition, the substantial phenotypic heterogeneity observed among 16p11.2 CNV carriers suggest that similar molecular perturbations may lead to divergent clinical outcomes, depending on genetic background, environmental exposures, and developmental context. As a result, therapeutic responses are likely to be similarly variable.

Future pharmacological strategies are therefore likely to benefit from network-based approaches that identify central nodes capable of influencing multiple phenotypes [85]. However, identifying such nodes remains challenging, and their modulation may have context-dependent or unintended effects across developmental stages. Rather than directly targeting individual genes, modulation of shared signaling pathways, such as ERK/MAPK or RhoA, may be more effective. However, the pleiotropic nature of these pathways presents substantial challenges for therapeutic specificity and safety. A key implication of the current findings is that the success of therapeutic intervention is likely to depend on developmental timing. Many of the cellular and circuit abnormalities observed in studies of 16p11.2 CNVs emerge during tightly defined periods of synapse formation, maturation, and circuit refinement. This suggests that certain interventions may only be effective within specific temporal windows. Outside these windows, they may fail to correct network abnormalities or may instead unmask compensatory adaptations [231]. Moreover, a key priority for future research is the development of more flexible and developmentally informed model systems. While current animal models have yielded valuable insight into the mechanisms of CNV biology, they rely on static, tightly controlled gene dosage changes that do not capture the dynamic and context-dependent nature of human neurodevelopment [232,233]. In particular, such models do not fully account for the influence of additional genetic variation and environmental factors, which are likely to play a critical role in shaping both developmental trajectories and treatment responses in human populations. Approaches that include developmental timing, such as longitudinal phenotyping or temporally controlled gene manipulation [122,234], may contribute towards our understanding of how early cellular disruptions translate into later outcomes. Together, these considerations highlight that translating mechanistic insights from 16p11.2 CNV research into effective therapeutic strategies will require careful integration of developmental timing, biological heterogeneity, and system-level complexity.

## 9. Conclusions

Collectively, the evidence reviewed here supports a model in which 16p11.2 CNVs disrupt development through dosage-sensitive perturbation of convergent signaling pathways that govern synaptic maturation, E/I balance, and circuit refinement. Rather than acting through a single dominant mechanism, multiple genes within the locus exert partially overlapping but mechanistically distinct effects on dendritic architecture, spine stability, and synaptic plasticity. The phenotypic consequences of these perturbations are strongly shaped by developmental timing, cell type, and circuit context. A key theme emerging across studies is that altered signaling does not uniformly impair neuronal function but instead biases activity-dependent plasticity, leading to miscoordination scaling from individual synapses to wider neural circuits. Circuits undergoing prolonged maturation and refinement may be particularly vulnerable, which may partially explain the variable manifestation of 16p11.2 CNVs in their impact on cognition and behavior. The differences between isolated gene-specific mechanisms and shared downstream phenotypes of this CNV highlight its inherent complexity. While no single pathway fully accounts for the disorder spectrum, convergence on synaptic and circuit-level dysfunction provides a unifying framework for understanding phenotypic heterogeneity and for guiding therapeutic exploration.

## 10. Outstanding Questions

1. To what extent can pathway-specific preclinical interventions be translated into clinically meaningful treatments for individuals with 16p11.2 CNVs?
2. Are the developmental constraints identified for 16p11.2 deletions and duplications shared across other recurrent neurodevelopmental CNVs?
3. How do mechanistically distinct gene-specific perturbations converge in shared synaptic and circuit-level phenotypes?

## Data Availability

No new data were created or analyzed in this study. Data sharing is not applicable to this article.

## Abbreviations

The following abbreviations are used in this manuscript:

NDD	Neurodevelopmental disorder
NPD	Neuropsychiatric disorder
CNV	Copy number variant
NAHR	Non-allelic homologous recombination
